# Genetic diversity of transmission-blocking vaccine candidates Pvs25 and Pvs28 in *Plasmodium vivax *isolates from Yunnan Province, China

**DOI:** 10.1186/1756-3305-4-224

**Published:** 2011-11-28

**Authors:** Hui Feng, Li Zheng, Xiaotong Zhu, Gege Wang, Yanyan Pan, Ying Li, Yimei Yang, Yahui Lin, Liwang Cui, Yaming Cao

**Affiliations:** 1 Department of Immunology, College of Basic Medical Sciences, China Medical University, Shenyang, China; 2 Institute of Pathology and Pathophysiology, China Medical University, Shenyang, China; 3 Department of Parasitology, College of Basic Medical Sciences, Dali Medical College, Dali, China; 4 Department of Microbiology and Parasitology, Institute of Basic Medical Sciences, Chinese Academy of Medical Sciences & School of Basic Medicine, Peking Union Medical College, Beijing, China; 5 Department of Entomology, Pennsylvania State University, University Park, Pennsylvania, USA

**Keywords:** *Plasmodium vivax*, TBVs, Pvs25, Pvs28, polymorphism

## Abstract

**Background:**

Transmission-blocking vaccines (TBVs) have been considered an important strategy for disrupting the malaria transmission cycle, especially for *Plasmodium vivax *malaria, which undergoes gametocytogenesis earlier during infection. Pvs25 and Pvs28 are transmission-blocking vaccine candidates for *P. vivax *malaria. Assessment of genetic diversity of the vaccine candidates will provide necessary information for predicting the performance of vaccines, which will guide us during the development of malaria vaccines.

**Results:**

We sequenced the coding regions of *pvs25 *and *pvs28 *from 30 *P. vivax *isolates from Yunnan Province, identifying five amino acid haplotypes of Pvs25 and seven amino acid haplotypes of Pvs28. Among a total of four mutant residues, the predominant haplotype of Pvs25 only had the I130T substitution. For Pvs28, a total of eight amino acid substitutions were identified. The predominant haplotype of Pvs28 had two substitution at positions 52 (M52L) and 140 (T140S) with 5-6 GSGGE/D tandem repeats at the end of fourth EGF-like domain. Most amino acid substitutions were common with previous reports from South Asian isolates. Although the nucleotide diversity of *pvs28 *(π = 0.0034 ± 0.0012) was significantly higher than *pvs25 *(π = 0.0013 ± 0.0009), it was still conserved when compared with the blood stage vaccine candidates.

**Conclusions:**

Genetic analysis revealed limited genetic diversity of *pvs25 *and *pvs28*, suggesting antigenic diversity may not be a particular problem for Sal I based TBVs in most *P. vivax*-endemic areas of China.

## Background

*Plasmodium vivax*, the most widespread species of human malaria parasites, is responsible for the majority of malaria cases outside of Africa, and the most prevalent form of relapsing malaria. Although not as lethal as *P. falciparum *malaria, *P. vivax *has caused substantial morbidity for human populations residing in Asia and South America [1,2]. Throughout the malaria control history, *P. vivax *malaria has displayed tremendous resilience to control efforts, which is in part due to two biological characteristics of this parasite. One is the formation of hypnozoites in the liver, which is responsible for relapses of the disease. The other is that *P. vivax *undergoes gametocytogenesis before manifestation of the disease symptoms, making transmission possible before treatment.

*P. vivax *is the predominant *Plasmodium *species in China, and in recent years, *P. vivax *cases accounted for more than 90% of all malaria cases [3]. As The Ministry of Health of China has set the goal of malaria elimination by 2020 [4], interruption of *vivax *malaria transmission is a major challenge. Yunnan is one of the two provinces that have year-round local transmission of *P. vivax *and *P. falciparum*. It is located in southwest China and borders Myanmar to the west and Laos and Vietnam to the south [5]. A total of 250,070 confirmed cases of *P. vivax *malaria and 44,465 cases of *P. falciparum *malaria have been reported between 1991 and 2006 in Yunnan [6]. In recent years, we have witnessed a significant decline of malaria cases in Yunnan [3]. A total of 32,566 confirmed cases of *P. vivax *malaria and 5,821 cases of *P. falciparum *malaria have been reported between 2006 and 2009 in Yunnan [3,7-9]. Most of the malaria cases are now clustered in counties located at the China-Myanmar border area [3,10]. Despite improvement in malaria situation in this region, malaria control along the borders is problematic, since reintroduction of malaria by human migration is still difficult to monitor. Thus, it is particularly important to develop effective strategies to control and eliminate malaria in this region.

Transmission-blocking vaccines (TBVs), which target the sexual stages of malaria parasites to prevent their further development within mosquitoes, are considered an important strategy for disrupting malaria transmission [11,12]. To date, several sexual stage antigens have been characterized, which showed excellent transmission blocking activities. These include the pre-fertilization antigens P48/45 and post-fertilization antigens P25 and P28. P25 and P28, are specifically expressed on *Plasmodium *zygote and ookinete surface [11], and they are essential for the survival of ookinetes in the mosquito midgut, and subsequent penetration of the midgut epithelium and transformation into oocysts [13]. Among the TBVs candidates, Pvs25 has received the most attention. Recombinant Pvs25 protein expressed in bacteria [14], yeasts [15,16] and baculovirus [17] exhibits strong immunogenicity and immune sera display significant transmission-blocking activity on the development of sporozoites [14] and oocysts [15-17]. The immunogenicity trials of recombinant Pvs25 have been undertaken in rhesus monkeys [18] and human volunteers [19,20]. A phase I clinical trial of Pvs25H vaccine in human volunteers has demonstrated that vaccine-induced antibodies have significant transmission-blocking activity [19]. It has been reported that antisera to recombinant Pvs25 and Pvs28 based on the Sal-I strain recognized the corresponding molecules expressed by field-isolated parasites in Thailand, and that these antisera blocked transmission of field isolates [15].

Polymorphisms represent a major impediment in vaccine design. An effective vaccine must either be based on conserved regions or incorporate multiple allelic forms of the antigen. As antigenic polymorphisms limit the immunogenicity and immunoreactivity of vaccines, it is necessary to understand antigenic variations in order to generate effective vaccines against natural *Plasmodium *infections [12]. Pvs25 and Pvs28 contained conserved structures, which are characterized by a secretory N-terminal signal sequence followed by four epidermal growth factor (EGF) domains and a glycosylphosphatidylinositol (GPI) anchor [21]. To date, genetic diversity of *pvs25 *and *pvs28 *genes has been surveyed in several Asian countries, including South Korea [22], Indonesia [23], Iran [24], India [25,26], Bangladesh [25], and Thailand [15]. In this study, we aim to analyze the genetic diversity of these candidate TBVs genes in parasite isolates from Yunnan Province, China.

## Methods

### Study areas

Yunnan Province is one of the most highly endemic regions in China with year-round transmission of *P. vivax *and *P. falciparum*. Since 2001, there has been a strong seasonal pattern of malaria incidence characterized by a peak of infection occurring from June to July and another peak from October to November [27]. Counties with higher malaria incidence rates are clustered in the border area and the Yuanjiang River Basin [6,27]. The predominant malaria vectors are *Anopheles minimus *and *Anopheles dirus *[28].

### Parasite collection

**Figure 1:**
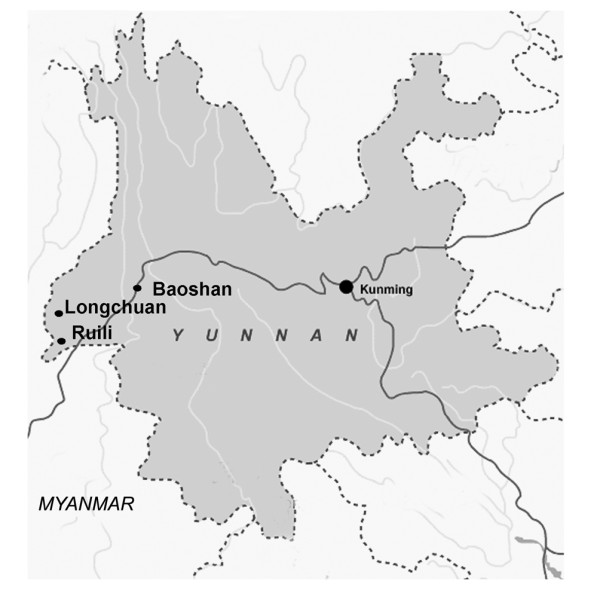
**Map of the Yunnan Province of China to show the sampling sites**.

A total of 30 parasite samples were collected in 2004 from three regions in Yunnan. All volunteers in this study were symptomatic patients diagnosed with *P. vivax *malaria using Giemsa stained thin smear examination by malaria clinic staff. After informed consent/assent was obtained from either adults or parents or legal guardians of children, ~100 μl of finger-prick blood were collected from each patient on filter papers. The patients were Yunnan local residents, 13 to 50 years old, living in Baoshan (10 individuals), Ruili (10 individuals) and Longchuan (10 individuals) (showed in Figure 1). Patients were then treated according to the Ministry of Health drug policy. This study protocol was approved by the Biomedical Research Ethics Review Board at China Medical University.

### Parasite DNA preparation and amplification

Three circles, 2.5 mm in diameter, were punched out from each dried blood spot and *Plasmodium *DNA was purified using the QIAamp DNA Mini kit (QIAGEN, Hilden, Germany) according to the manufacturer's protocol. DNA was eluted in 60 μl of H_2_O. *pvs25 *(GenBank: AF083502.1) and *pvs28 *(GenBank: AF083503.2) of the Salvador I (Sal I) strain were used as reference sequences. The full-length open reading frames (ORFs) of *pvs25 *and *pvs28 *were amplified using the following primer pairs: *pvs25*F (5'-CACTTAGCCAAAATGAACTC-3') and *pvs25 *R (5'-AAAGGACAAGCAGGATGATA-3') for *pvs25*; *pvs28*F (5'-CTACCACAGCTTGCTGTTCC-3') and *pvs28*R (5'-TGACATCATGAAGAAGGCG-3') for *pvs28*. The PCR reaction contained 2 μl of 10 × KOD-Plus buffer, 2 μl of 2 mM dNTPs, 0.8 μl of 25 mM MgSO_4_, 1.0 μl of 10 μM of each primer, 0.2 units of KOD-Plus DNA polymerase (Toyobo, Osaka, Japan), and 0.5 μl genomic DNA template in a final volume of 20 μl. Amplification conditions were as follows: initial denaturing at 94°C for 2 min, 35 cycles of 94°C for 15 sec, 56°C (for *pvs28*) or 54°C (for *pvs25*) for 15 sec, and 68°C for 1 min, and a final extension at 68°C for 5 min. PCR products were cleaned up using Exonuclease I and Shrip alkaline phosphatase treatment according to the manufacturer's instructions (USB, CA, USA).

### DNA sequencing

*Pvs25 *and *pvs28 *ORFs were sequenced using the ABI Prism^® ^BigDye™ Terminator cycle sequencing kit (Applied Biosystems, Foster City, CA, USA) on an ABI PRISM^® ^310 Genetic Analyzer (Applied Biosystems, Foster City, CA, USA). Sequencing primers are: *pvs25*F (5'-GAAACCCTAGGCAAAGCATG-3') and *pvs25*R (5'- GGGACTTTGCCA ATAGCACA-3') for *pvs25*, and *pvs28*F (5'-AACTGTGGAGACTACGCTG-3') and *pvs28*R (5'-ATATTACAAGAGCACATGGTG-3') for *pvs28*.

### Statistical Analysis

The ORFs of *pvs25 *and *pvs28 *were aligned with the reference sequences using the CLUSTAL W program [29] with manual corrections. Nucleotide diversity (*π*) and its standard error (SE) were calculated with the Jukes and Cantor method using MEGA 4.0. The number of haplotypes (*h*), haplotypes diversity (*Hd*), segregating sites (*S*) were computed using the options available in DnaSP 5.0 [30].

## Results

### Sequence polymorphisms of the *pvs25 *and *pvs28 *genes

*Pvs25 *and *pvs28 *genes were successfully amplified in 30 *P. vivax *samples obtained from Yunnan. The sequence of *pvs25 *(645 bp) and *pvs28 *(712 bp) were determined by direct sequencing of the PCR products. The 30 *pvs25 *sequences contained four polymorphic nucleotide sites (C103A, G289C, T389C, and C391A), which resulted in four amino acid substitutions (L35M, E97Q, I130T, and Q131K). These substitutions resulted in a total of five amino acid haplotypes, hitherto referred to as Pvs25-I, II, III, IV and V (Table 1). The predominant amino acid haplotype of Pvs25 only had the I130T substitution.

**Table 1:** Amino acid substitutions of Pvs25 from Yunnan *P. vivax *isolates

Haplotypes (number)	EGF-1	EGF-2	EGF-3	EGF-3
	35	97	130	131
Sal-I	L	E	I	Q
Pvs25-I (n = 15)	•	•	T	•
Pvs25-II (n = 8)	•	•	T	K
Pvs25-III (n = 5)	•	Q	T	•
Pvs25-IV (n = 1)	•	Q	T	K
Pvs25-V (n = 1)	M	•	T	K

EGF, epidermal growth factor-like domain. • indicates identical amino acid residues compared to the Salvador I strain.

The 30 *pvs28 *sequences had nucleotide polymorphisms at 13 sites, which included four synonymous substitutions (T346C, T558C, C573T, and G579A) and eight nonsynonymous substitutions (G40T, A154C, T292A, G313A, T346G, C419G, G536A, and C666G), which resulted in eight amino acid substitutions (V14L, M52L, L98I, E105K, L116V, T140S, G179E, and I222M). A total of seven amino acid haplotypes were identified: Pvs28-I, II, III, IV, V, VI and VII (Table 2). The predominant amino acid haplotype of Pvs28 had two amino acid substitutions at positions 52 (M52L) and 140 (T140S). Compared with Sal-I strain, we found the majority of clones had six copies of GSGGE/D tandem repeats at the end of fourth EGF-like domain. Only one clone with Pvs28-I haplotype had five copies of repeat regions.

**Table 2:** Amino acid substitutions of Pvs28 from Yunnan *P. vivax *isolates

Haplotypes (number)	SS	EGF-1	EGF-2	EGF-2	EGF-3	EGF-3	RR	RR	THR
	14	52	98	105	116	140	179	#	222
Sal-I	V	M	L	E	L	T	G	6	I
Pvs28-I (n = 19)	•	L	•	•	•	S	•	5/6	•
Pvs28-II (n = 4)	•	•	I	•	V	•	•	6	•
Pvs28-III (n = 2)	L	•	•	K	V	•	•	6	•
Pvs28-IV (n = 2)	•	L	•	•	•	•	•	6	•
Pvs28-V (n = 1)	•	•	•	•	•	•	•	6	M
Pvs28-VI (n = 1)	•	•	•	•	V	•	•	6	M
Pvs28-VII (n = 1)	•	L	•	•	•	•	E	6	•

SS, secretary signal sequence; EGF, epidermal growth factor-like domain; RR, repeat regions, # indicates the number of the GSGGE/D repeats; THR, the C terminal hydrophobic region.

• indicates identical amino acid residues compared to the Salvador I strain.

### Compared with the worldwide isolates

Compared with previous reports of worldwide isolates, Pvs25-I haplotype showed 100% identity with isolates from Vietnam (GenBank: ABG29072.1), North Korea (GenBank: AAV33640.1) [23], Thailand (GenBank: AB091730.1) [15] and Mexico (GenBank: ABS70906.1-ABS70935.1) [31]. The Pvs25-II haplotype was similar to some isolates from Bangladesh (GenBank: BAA94348.1-BAA94350.1) [25] and Thailand (GenBank: BAC66001.1) [15]. The Pvs25-III haplotype was common with isolates from Iran (GenBank: ACJ54129.1, ACJ54128.1) [24], Thailand (GenBank: BAC66003.1) [15], Indonesia (GenBank: AAV33639.1) [23], India (GenBank: BAA94346.1) [25] and Bangladesh (GenBank: BAA94347.1) [25]. Only Pvs25-IV was unique in China. Like in some South Korean isolates, position 35 was also polymorphic, but the substitution is L35M in the Yunnan isolate as compared to L35P in South Korean isolates (GenBank: ADJ18839.1) [22].

The Pvs28-I haplotype in Yunnan isolates was also common in isolates from Iran (GenBank: ACJ54136.1) [24] and Bangladesh (GenBank: BAA94352.1, BAA94353.1, BAA94371.1, BAA94362, BAA94363) [25]. The Pvs28-II haplotype was similar with those from Thailand (GenBank: BAC66011.1) [15] and Bangladesh (GenBank: BAA94370.1, BAA94369.1) [25]. Parasite isolates from Yunnan contained some region-specific haplotypes. Haplotype Pvs28-III with a new V14L substitution was found only in one isolate. The remaining haplotypes (Pvs28-IV, V and VI) were only identified in the Yunnan isolates. The G179E mutation was only found in South Korean (GenBank: ADJ18881.1) [22] and Chinese isolates.

The Pvs25 and Pvs28 amino acid substitutions identified from worldwide isolates including those from China are summarized in Tables 3 and 4. Among a total of 17 variant amino acids in Pvs25 from worldwide isolates, I130T was the most common amino acid substitution from American and Asian isolates. The Q87K substitution was common in American isolates such as those from Brazil [23] and Mexico [31], and Western Asian isolates such as those from Iran [24] and Turkey, but not detected in South and East Asian isolates. Amino acid residue E97Q was detected in some Asian isolates such as those from Iran [24], Bangladesh [25], Thailand [15], India [25,26], Indonesia [23], South Korea [22], Central China [32] and Yunnan, but rare in American isolates. Compared with previous studies from Central China isolates, amino acid residue I130T was the major amino acid substitution in Yunnan, Zhejiang and Hubei isolates [32]. The amino acid residue Q131K was not detected in Zhejiang and Hubei isolates [32], but it was very common in South Asian isolates such as those from Bangladesh [25], Thailand [15], India [26], and also in Yunnan isolates.

**Table 3:** Amino acid variations of Pvs25 among worldwide isolates

	SS	EGF-1		EGF-2		EGF-3						EGF-4					THR	
												
Country	2	35	38	87	97	130	131	132	137	138	149	170	174	183	196	198	199	Ref.
Sal-I	N	L	M	Q	E	I	Q	S	C	A	K	C	E	E	S	S	V	AAC99769
Iran	•	•	•	Q/K	E/Q	T	•	•	•	•	•	•	•	•	•	•	•	[24]
Mexico	•	•	•	Q/K	•	I/T	•	•	•	•	•	•	•	•	•	•	•	[31]
Brazil	•	•	•	K	•	•	•	•	•	•	•	•	•	•	•	•	•	[23]
Colombia	•	•	•	K	•	•	•	•	•	•	•	•	•	•	•	•	•	[23]
Venezuela	•	•	T	K	•	•	•	•	•	•	•	•	•	•	•	•	•	[23]
Mauritania	•	•	•	K	•	T	•	•	•	•	•	•	•	•	•	•	•	[23]
Turkey	•	•	•	K	•	T	•	•	•	•	•	•	•	•	•	•	•	ABG29073
PNG	•	•	•	•	•	T	K	R	•	•	N	•	•	•	•	•	•	[23]
Nicaragua	•	•	•	•	•	•	•	•	•	•	•	R	•	•	•	•	•	[23]
India	•	•	•	•	Q	T	•	•	•	•	•	•	•	•	•	•	•	[25]
India	•	•	•	•	E/Q	T	Q/K	•	C/W	A/G	•	•	E/K	E/K	S/F	S/T	V/E	[26]
Bangladesh	•	•	•	•	E/Q	T	Q/K	•	•	•	•	•	•	•	•	•	•	[25]
Vietnam	•	•	•	•	•	T	•	•	•	•	•	•	•	•	•	•	•	ABG29072
Indonesia	•	•	•	•	Q	T	•	•	•	•	•	•	•	•	•	•	•	[23]
Thailand	•	•	•	•	E/Q	T	Q/K	•	•	•	•	•	•	•	•	•	•	[15]
N Korea	•	•	•	•	•	T	•	•	•	•	•	•	•	•	•	•	•	[23]
S Korea	N/D	•	•	•	E/Q	T	•	•	•	•	•	•	•	•	•	•	•	[22]
China (ZJ/HB)	•	•	•	•	E/Q	T	•	•	•	•	•	•	•	•	•	•	•	[32]
China (YN)	•	L/M	•	•	E/Q	T	Q/K	•	•	•	•	•	•	•	•	•	•	This study

SS, secretary signal sequence; EGF, EGF-like domain; THR, the C terminal hydrophobic region. Ref: references. ZJ/HB and YN represented isolates from Zhejiang/Hubei and Yunnan Provinces in China, respectively. • indicates identical amino acid residues compared to the Salvador I strain.

**Table 4:** Amino acid variations of Pvs28 among worldwide isolates

	SS		EGF-1		EGF-2								EGF-3				EGF-4		RR					THR		
																	
Country	0 0 5	0 1 4	0 5 2	0 5 3	0 6 5	0 7 9	0 8 1	0 8 7	0 9 5	0 9 8	1 0 5	1 0 6	1 1 0	1 1 3	1 1 6	1 4 0	1 5 9	1 7 9	1 9 1	2 0 8 *	2 1 0 #	2 1 4 $	GSGGE/D	2 2 1 &	2 2 2 @	Ref.
Sal-I	H	V	M	A	T	V	A	D	G	L	E	V	N	N	L	T	K	G	G	D	G	S	6	V	I	AAC99770
Iran	•	•	L	•	T/K	•	•	•	•	•	•	•	•	•	•	S	•	•	•	•	•	•	4-6	•	•	[24]
Mexico	•	•	L	•	•	•	•	N	•	•	•	•	Y	•	•	S	•	•	•	•	•	•	5-6	•	•	[31]
Bangladesh	•	•	M/L	•	T/K	•	•	•	•	L/I	•	•	•	N/S	L/V	T/S	K/ R	•	•	•	•	•	5-7	•	I/ M	[25]
Thailand	•	•	M/L	A/V	T/K	•	A/V	•	G/N	L/I	E/K	V/E	•	•	L/V	T/S	•	•	•	•	•	•	5-7	•	•	[15]
India	•	•	L	•	K	•	•	•	•	•	•	•	•	•	•	S	•	•	•	•	•	•	4	•	•	[25]
India	H/T/Y	•	M/L	A/V	T/K	V/E	•	•	•	L/I	E/K	•	•	•	L/V	T/S	•	•	D	D/G	G/R	S/ T	3-6	V/L	I/M	[26]
S Korea	•	•	L	•	•	•	•	•	•	•	•	•	•	•	•	S	•	G/ E	G/ R	•	•	•	6	•	•	[22]
China (YN)	•	V/L	M/L	•	•	•	•	•	•	L/I	E/K	•	•	•	L/V	T/S	•	G/E	•	•	•	•	5-6	•	I/M	This study

SS, secretary signal sequence; EGF, EGF-like domain; RR, repeat regions; THR, the C terminal hydrophobic region. Ref.: references. YN represented isolates from Yunnan Provinces in China. • indicates identical amino acid residues compared to the Salvador I strain.

*, #, $, &, @ represented amino acid residues 210, 212, 216, 223, 224 reported in reference 26, respectively.

Among a total of 24 amino acid substitutions in Pvs28 from worldwide isolates, M52L and T140S were the most common substitutions from different geographic regions [15,24-26,31]. The amino acid residues L98I and L116V were very common in South Asia isolates such as those from Bangladesh [25], Thailand [15], India [26], and also in Yunnan isolates. The amino acid residue T65K was not identified in isolates from Yunnan and South Korea [22], but was common in those from Bangladesh [25], Thailand [33], India [25,26] and Iran [24].

### Genetic diversity of *pvs25 *and *pvs28*

There were a total of four polymorphic sites that generated five haplotypes in *pvs25*. There were a total of 12 polymorphic sites that yielded eight haplotypes in *pvs28*. Nucleotide diversity of *pvs28 *(π = 0.0034 ± 0.0012) was significantly higher than that of *pvs25 *(π = 0.0013 ± 0.0009) (*p *< 0.05) (Table 5).

**Table 5:** Nucleotide diversity of the *pvs25 *and *pvs28 *genes

Gene	n	*S*	*h*	Hd	*π ± *SE
*pvs25*	30	4	5	0.671	0.0013 ± 0.0009
*pvs28*	30	12	8	0.556	0.0034 ± 0.0012

n, number of sequences samples; *S*, number of polymorphic sites; *h*, number of haplotypes; Hd, haplotype diversity; π, nucleotide diversity; nucleotide diversity with its standard error (SE) were computed with Jukes-Cantor correction using MEGA 4.0.

## Discussion

In this study, we assessed the level of genetic diversity of *pvs25 *and *pvs28 *genes in *P. vivax *isolates from Yunnan Province, China. A total of four and eight amino acid substitutions have been identified in Pvs25 and Pvs28, respectively. Compared with *P. vivax *isolates from previous studies conducted in Asia, our results showed that the majority of the variant amino acids of Pvs25 and Pvs28 detected in Yunnan isolates were shared with those reported in isolates from South Asia (Bangladesh, Thailand and India) [15,25]. Because of its location in southwest China, Yunnan isolates had mutant residues characteristic of South Asian isolates, such as mutant residues E97Q and Q131K in Pvs25, as well as L98I and L116V in Pvs28. It suggested that some parasite haplotypes may have different geographic distributions.

One of the major obstacles to the development of an effective malaria vaccine is the genetic polymorphism of many of the genes in natural parasite populations that otherwise would be promising vaccine candidates. A number of studies have shown that TBVs candidates have limited polymorphism compared to antigens expressed in asexual stage parasites [34,35]. Our data showed that *pvs25 *(π = 0.0013) had a much lower level of diversity than *P. vivax *blood stage proteins DBP (π = 0.0086-0.0184) [36] and MSP1 (π = 0.1193-0.2055) [37]. In addition, compared with *P. falciparum *sexual stage proteins *pfs25 *(π = 0.0035) [34], the lower genetic diversity of *pvs25 *suggested that genetic polymorphism of *pvs25 *was limited in Yunnan isolates. Although the nucleotide diversity of *pvs28 *was significantly higher (π = 0.0034) than that of *pvs25*, it was still conserved when compared with the blood stage vaccine candidates. Although direct sequencing of the PCR products in our study might underestimate the genetic diversity of these two genes, the effect should be minor since our study areas are malaria hypoendemic and mixed infections should not be prevalent. The limited diversity of sexual stage antigens such as P25 and P28 is perhaps attributed to the expression of these proteins in mosquito stages, which should avoid immune selection in the humans.

It is very important to evaluate the effect of genetic polymorphism of Pvs25 and Pvs28 on the efficacy of Sal-I based TBVs in different malaria epidemiological areas. The transmission-blocking assays conducted in Thailand clearly demonstrated that antisera to recombinant Pvs25 and Pvs28 based on the Sal-I strain of *P. vivax *recognized corresponding molecules expressed by field-isolated parasites in Thailand. Our data showed limited genetic diversity of *pvs25 *and *pvs28 *in the Yunnan isolates. The major amino acid haplotype of Pvs25 from this region was also shared with that from Thailand isolates, it provids a good prospect on implication of TBVs in China malaria areas.

Altogether, our study offers a first glimpse of the genetic diversity of two TBVs candidate antigens in Yunnan Province, China. Yet, this study only offered limited information of *P. vivax *parasites from Yunnan. Further studies on a larger sample scale and comparison analysis with Central China isolates will certainly improve our understanding of genetic polymorphisms of TBVs candidates in the malaria endemic areas of China.

## Conclusions

TBVs are one of the important strategies for controlling malaria transmission. A key point in vaccine optimization is to understand the extent of genetic diversity of candidate antigens. The TBVs candidates Pvs25 and Pvs28 from Yunnan isolates showed limited genetic diversity. Further studies encompassing larger malaria endemic areas are needed to provide a thorough evaluation of situation in China.

## Competing interests

The authors declare that they have no competing interests.

## Authors' contributions

YC conceived of the study and helped to draft the manuscript. YY collected the blood spots specimens. HF carried out the studies, statistical analysis and drafted the manuscript. LZ, XZ, GW, YP, YL, YL participated in the molecular genetic studies and sequence alignment. LC helped with statistical analysis and critically revised the manuscript. All authors contributed to the writing of the manuscript and approved the submitted version of the manuscript.
